# An administrative model for benchmarking hospitals on their 30-day sepsis mortality

**DOI:** 10.1186/s12913-019-4037-x

**Published:** 2019-04-11

**Authors:** Jennifer L. Darby, Billie S. Davis, Ian J. Barbash, Jeremy M. Kahn

**Affiliations:** 1 CRISMA Center, Department of Critical Care Medicine, University of Pittsburgh School of Medicine, Pittsburgh, PA USA; 2 Division of Pulmonary, Allergy, and Critical Care, University of Pittsburgh School of Medicine, Pittsburgh, PA USA; 3 Department of Health Policy and Management, University of Pittsburgh Graduate School of Public Health, Pittsburgh, PA USA; 4 Critical Care Medicine and Health Policy & Management, University of Pittsburgh, Scaife Hall Room 602-B, 3550 Terrace Street, Pittsburgh, PA 15221 USA

**Keywords:** Sepsis, Intensive care, Critical care, Mechanical ventilation, Performance, Quality, Outcomes

## Abstract

**Background:**

Given the increased attention to sepsis at the population level there is a need to assess hospital performance in the care of sepsis patients using widely-available administrative data. The goal of this study was to develop an administrative risk-adjustment model suitable for profiling hospitals on their 30-day mortality rates for patients with sepsis.

**Methods:**

We conducted a retrospective cohort study using hospital discharge data from general acute care hospitals in Pennsylvania in 2012 and 2013. We identified adult patients with sepsis as determined by validated diagnosis and procedure codes. We developed an administrative risk-adjustment model in 2012 data. We then validated this model in two ways: by examining the stability of performance assessments over time between 2012 and 2013, and by examining the stability of performance assessments in 2012 after the addition of laboratory variables measured on day one of hospital admission.

**Results:**

In 2012 there were 115,213 sepsis encounters in 152 hospitals. The overall unadjusted mortality rate was 18.5%. The final risk-adjustment model had good discrimination (C-statistic = 0.78) and calibration (slope and intercept of the calibration curve = 0.960 and 0.007, respectively). Based on this model, hospital-specific risk-standardized mortality rates ranged from 12.2 to 24.5%. Comparing performance assessments between years, correlation in risk-adjusted mortality rates was good (Pearson’s correlation = 0.53) and only 19.7% of hospitals changed by more than one quintile in performance rankings. Comparing performance assessments after the addition of laboratory variables, correlation in risk-adjusted mortality rates was excellent (Pearson’s correlation = 0.93) and only 2.6% of hospitals changed by more than one quintile in performance rankings.

**Conclusions:**

A novel claims-based risk-adjustment model demonstrated wide variation in risk-standardized 30-day sepsis mortality rates across hospitals. Individual hospitals’ performance rankings were stable across years and after the addition of laboratory data. This model provides a robust way to rank hospitals on sepsis mortality while adjusting for patient risk.

**Electronic supplementary material:**

The online version of this article (10.1186/s12913-019-4037-x) contains supplementary material, which is available to authorized users.

## Background

Sepsis is a leading cause of in-hospital mortality and a major driver of health care spending in developed nations [1]. Several evidence-based practices for sepsis exist, including adequate control of the infectious source, early administration of appropriate antibiotics, and early administration of intravenous fluids to support intravascular volume [2]. However, hospitals deliver these treatments inconsistently, leading to excess morbidity and mortality [3, 4]. In response to this persistent quality gap, health systems and governments have developed large scale strategies to improve sepsis care both through traditional clinically-oriented quality improvement [5] and through health policies designed to incentivize quality improvement at the regional and national level [6, 7].

Understanding the impact of these efforts and providing hospitals with feedback on their quality of care in patients with sepsis requires a robust method for assessing hospital-specific mortality rates. Such a method would ideally use widely available data that is readily accessible across hospital systems and must effectively account for individual patients’ variation in risk of mortality. At the same time, mortality-based performance measures should not adjust for variation in treatment practices that may modify the risk of mortality, which are reflective of hospital quality.

To address this need, we used a state-wide Pennsylvania discharge database that captures administrative claims data along with a selection of laboratory data to create a novel method to adjust for individual patients’ severity of illness on presentation in order to meaningfully compare sepsis outcomes across hospitals.

## Methods

### Study design and data

We conducted a retrospective cohort study of patients with sepsis admitted to non-federal general acute care hospitals in the Commonwealth of Pennsylvania in the United States during calendar years 2012 and 2013. First, we developed a de novo risk-adjustment model using 2012 administrative data. Next, we examined the construct validity of our model by examining the stability of hospital rankings over time (comparing the 2012 administrative model to the 2013 administrative model) and after addition of clinical laboratory variables (comparing the 2012 administrative model to a 2012 clinical model with both administrative and laboratory data). In this context, a valid administrative model would produce relatively stable performance estimates over time (i.e. with few exceptions, hospitals that are high performers one year would be high performers the next year). A valid administrative model would also yield performance estimates that are similar to those estimated from a more granular clinical model which better accounts for variation in risk.

We used the Pennsylvania Health Care Cost Containment Council (PHC4) database. PHC4 collects administrative data on all hospital admissions in Pennsylvania and makes them available for research, including both demographic information and *International Classification of Diseases—version 9.0—Clinical Modification* (ICD-9-CM) diagnosis and procedure codes. Unlike most administrative claims-based data sets, these data also contain a selection of laboratory values obtained on the day of admission, enabling us to create a clinical model in addition to the standard administrative model [8]. We augmented these data with the Pennsylvania Department of Health vital status records to capture post-discharge mortality.

### Patients and hospitals

All encounters for patients meeting the “Angus” definition of sepsis—either an explicit ICD-9-CM code for sepsis or co-documentation of ICD-9-CM codes for an infection and an organ dysfunction—were eligible for the study [9, 10]. We chose the Angus definition because it is the broadest administrative definition of sepsis and has undergone rigorous clinical validation (10). We excluded admissions to non-short term and non-general acute care hospitals as these hospitals were not the focus of our study. We also excluded admissions less than 20 years of age, admissions for which gender or age were missing, and admissions at hospitals that were not continuously open and admitting patients for the duration of the study period. To maintain independence of observations, if a single patient had multiple encounters within a study year, then we randomly included a single encounter per year.

### Base model for risk-adjusted mortality

We first created a base logistic regression model for risk-adjusted mortality using exclusively risk-adjustment variables that are available in administrative data. The primary outcome variable for this model was all-cause mortality within 30 days of the admission date, as determined using the Pennsylvania vital status records. The model was based on five categories of risk-adjustment variables hypothesized to be associated with sepsis outcomes based on prior work [9, 11, 12]: demographics, admission source, comorbidities, organ failures present on admission, and infection source.

Demographic variables were obtained directly from the claims and included age and gender. Gender was modeled as an indicator covariate, and age was modeled as a linear spline by age quintile. Admission source was obtained directly from the claims and modelled as an indicator covariate defined as admission through the emergency department versus admission from another source. Comorbidities were defined using ICD-9-CM codes in the manner of Elixhauser [13] and modelled as indicator covariates. Organ failures present on admission were defined in the manner of Elias [12] and modelled as indicator covariates. For comorbidities and organ failures present on admission, we excluded from the model any designation that had less than a 1% prevalence in our sample population.

Infection source was modeled as hierarchical infection categories in which we assigned each patient an infectious source category identified using ICD-9-CM diagnosis codes (see Additional file 1: Table S1). We created the categories from the Angus sepsis definition [9] which we further divided into 12 groups: septicemia, bacteremia, fungal infection, peritoneal infection, heart infection, upper respiratory infection, lung infection, central nervous system infection, gastrointestinal infection, genitourinary infection, skin infection, and other infection source. For patients with multiple ICD-9-CM codes indicating multiple infection sources, we assigned them the single infection source category associated with the highest unadjusted mortality. In ranking the infectious sources based on their unadjusted mortality, we used 2011 data in order to avoid model overfitting. The final variable was modelled as a series of mutually exclusive indicator covariates with upper respiratory infection as the reference category.

### Augmented mortality model including laboratory variables

We next created an augmented logistic regression model for risk-adjusted mortality using all of the variables from the base model plus selected laboratory values obtained on the day of admission. The list of available laboratory values including their units, frequency, averages, and ranges is available in Additional file 1: Table S2 and S3. Values outside the plausible range, such as negative data points for non-calculated laboratory values, were recoded as missing.

We used a multi-step process to determine not only which lab variables to include in our model but also their functional forms. First, we used locally weighted scatterplot smoothing to visually assess inflection points in the relationship between each numeric laboratory value and 30-day mortality [14]. Based on visual inspection of these plots and standard reference values from our hospital’s laboratory, we categorized each variable into between two and five categories, with one category representing a normal result and the other categories representing non-normal extremes: very low, low, high, and very high. For arterial pH and arterial pCO_2_, which are interdependent, we performed an additional step in which we created a single combined variable for which the categories were permutations of the non-normal categories defined for pH and pCO_2_, respectively, as previously performed [15].

For each patient, we assigned an appropriate category for every laboratory test based on the reported result. If the patient had more than one result available for a given laboratory test, we selected the value that would be included in the category associated with a higher mortality rate. When a laboratory test result was missing, we assumed it to fall into the normal range and assigned the normal category, as is standard in physiological risk-adjustment models [15].

Next, we used Bayesian information criterion (BIC)-based stepwise logistic regression to identify the laboratory value covariates to be included in the model. This regression included all the covariates in the claims-based model. Laboratory values that did not contribute to a maximal BIC were excluded from the final model. Each laboratory value’s categories were assessed in the BIC regression as a group and ultimately either included in or excluded from the model as a group, so as not to partially remove categories for a given laboratory value. Laboratory values deemed contributory by the BIC regression entered the final model as categorical variables with the normal category as the reference group.

### Risk-standardized mortality rates

Based on these models we use mixed-effects logistic regression to create risk-standardized hospital-specific 30-day mortality rates. These rates account for variation in both risk and reliability across hospitals: they account for variation in risk in that they control for the different baseline characteristics of sepsis patients across hospitals; they account for reliability in that the rates for small hospitals, which are more susceptible to random variation than rates for large hospitals, are adjusted toward the state-wide mean [16].

We calculated hospital-specific risk adjusted mortality rates by dividing each hospital’s predicted mortality (using the base model plus a hospital-specific random effect) by each hospital’s expected mortality (using the base model without a hospital-specific random effect), generating a risk-standardized mortality ratio. Multiplying the risk-standardized mortality ratio by the mean 30-day mortality of the state-wide sample yielded a hospital-specific risk-standardized mortality rate.

We performed this process separately for 2012 and 2013 without laboratory data and then again for 2012 with laboratory data, resulting in three sets of hospital-specific mortality rates: 2012 administrative rates, 2013 administrative rates, and 2012 clinical rates.

### Analysis

For all models we assessed discrimination, using the C-statistic, and calibration, using the slope and intercept of regression lines fit to the calibration plots. We assessed the validity of our administrative model by examining the consistency of hospital rankings over time and with the addition of laboratory data. As noted above, we assumed that a valid model would yield hospital rankings that did not markedly change between years or after the addition of laboratory values. We generated scatter plots to compare the hospital-specific risk-standardized mortality rates between the 2012 and 2013 administrative rates; and between the 2012 administrative and clinical rates, calculating a coefficient of determination. Additionally, for each of the three sets of hospital-specific mortality rates, we calculated performance quintiles, with the outer quintiles representing the highest and lowest performing 20% of hospitals, respectively. We compared the composition of the quintiles between the 2012 and 2013 administrative rates and then between the 2012 administrative and clinical rates. We considered hospital movement of one quintile or less between comparison groups to be a marker of stability.

Data management and analysis was performed using Stata version 14.0 (StataCorp, College Station, Texas). All aspects of this work were reviewed and approved by the University of Pittsburgh institutional review board.

## Results

### Patients and model development

**Fig. 1:**
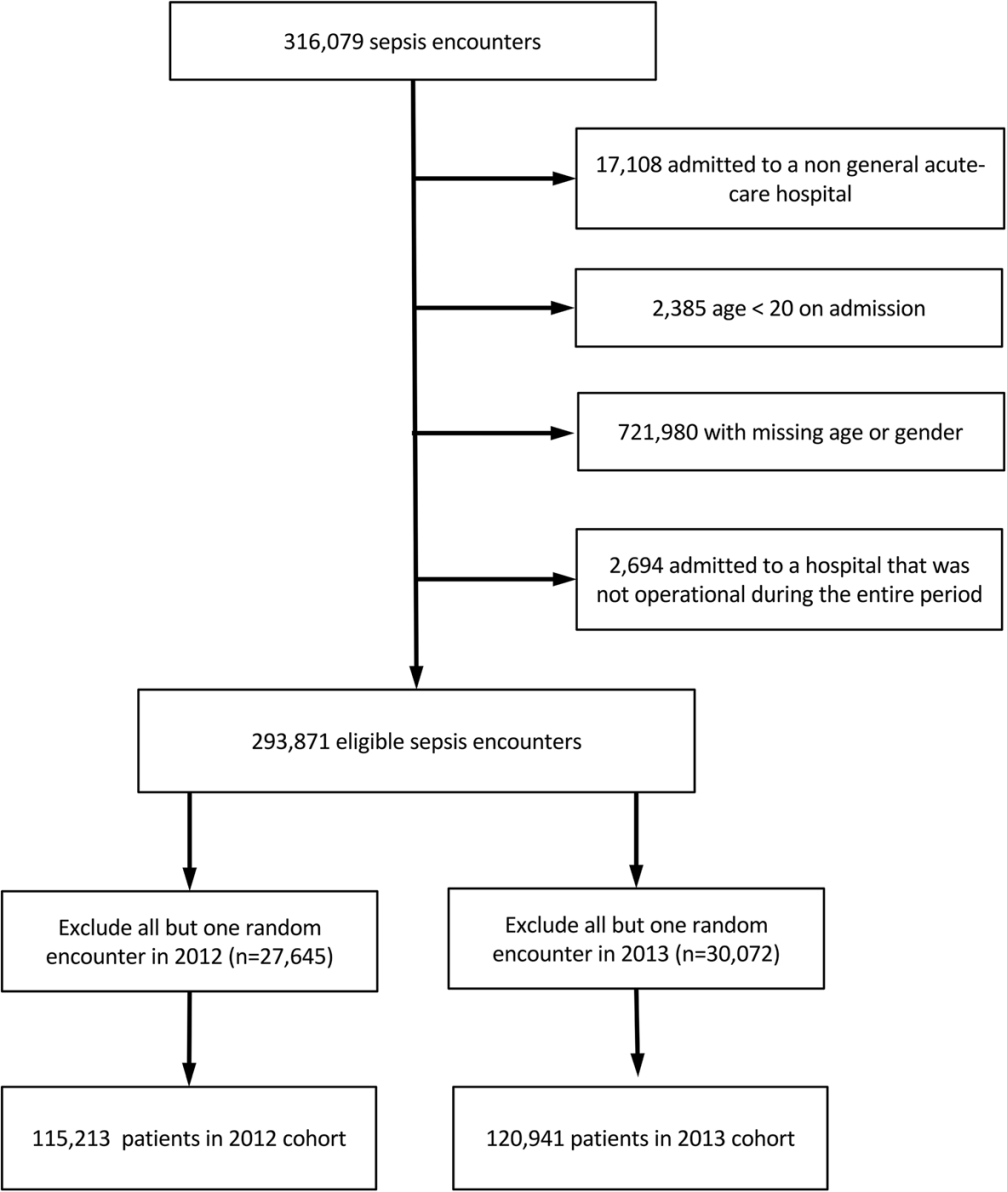
Patient flow diagram

A total of 236,154 patients met our final inclusion criteria: 115,213 in 2012 and 120,941 in 2013 (Fig. 1). These patients were admitted to 152 different acute care hospitals. Patient characteristics stratified by year are shown in Table 1. In both years average age was over 70 and a large percentage of patients were admitted through the emergency department. The most common comorbidity was hypertension (58.2% in 2012 and 57.4% in 2013) followed by fluid and electrolyte disorders, renal disease, diabetes, congestive heart failure, and chronic pulmonary disease. The most common organ failure on admission was renal failure (48.2% in 2012 and 49.1% in 2013) followed by cardiovascular failure. Unadjusted 30-day mortality was 18.5% in 2012 and 18.2% in 2013.

**Table 1:** Patient characteristics by year represented as percentage of patients with given characteristic, except for age which is represented as a numerical average

Characteristic	2012 (*n* = 115,213)	2013 (*n* = 120,941)
Age (years)	70.5	70.3
Female	52.2%	52.0%
Admitted through the Emergency Department	80.5%	80.3%
Comorbidities		
Congestive heart failure	21.2%	21.7%
Valvular disease	7.0%	7.1%
Pulmonary circulation disease	5.3%	5.5%
Peripheral vascular disease	9.3%	8.9%
Paralysis	4.6%	4.5%
Neurological disorder	12.6%	12.6%
Chronic pulmonary disease	25.5%	25.9%
Diabetes w/o chronic complications	24.8%	24.3%
Diabetes w/ chronic complications	8.3%	8.4%
Hypothyroidism	15.2%	15.3%
Renal disease	27.0%	26.2%
Liver disease	5.0%	4.8%
Lymphoma	1.6%	1.6%
Metastatic cancer	4.6%	4.4%
Solid tumor w/o metastasis	3.5%	3.6%
Rheumatoid arthritis/collagen disease	3.3%	3.3%
Coagulopathy	18.0%	17.5%
Obesity	11.9%	12.2%
Weight Loss	13.0%	12.6%
Fluid and electrolyte disorder	54.0%	55.3%
Chronic blood loss anemia	1.3%	1.2%
Deficiency anemia	26.5%	25.3%
Alcohol abuse	4.2%	4.2%
Drug abuse	2.4%	2.6%
Psychotic disorder	6.2%	6.1%
Depression	11.8%	11.8%
Hypertension	58.2%	57.4%
Organ failures present-on-admission		
Septic shock	7.8%	8.3%
Respiratory failure	13.6%	14.4%
Cardiovascular failure	19.2%	19.8%
Renal failure	48.2%	49.1%
Hepatic failure	2.4%	2.4%
Hematologic failure	12.4%	12.0%
Metabolic failure	10.2%	11.2%
Neurologic failure	1.9%	1.9%
30-day unadjusted mortality	18.5%	18.2%

**Fig. 2:**
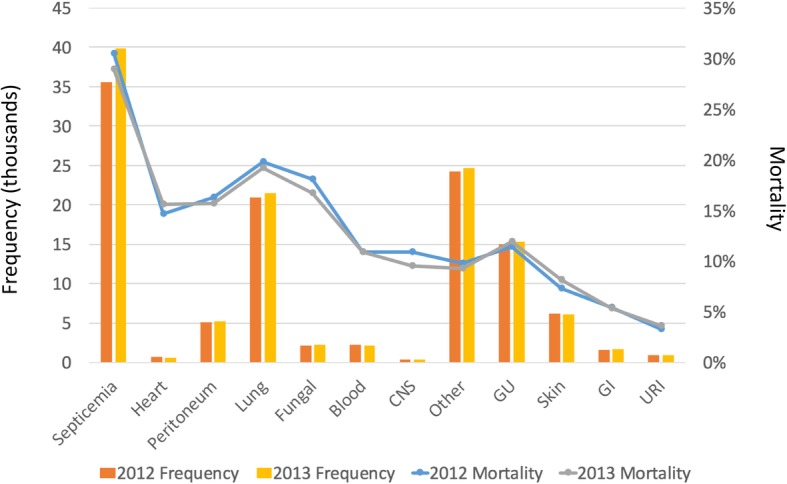
Number of patients assigned to each hierarchical infection category and the 30-day mortality rate for each hierarchical infection category, stratified by year. Abbreviations: CNS = central nervous system; GU = genitourinary tract; GI = gastrointestinal tract; URI = upper respiratory infection

The hierarchical infection categories along with each category’s mortality rate and the number of patients who were placed into that infection category are shown in Fig. 2. In both years, septicemia was the most prevalent category (30.9% in 2012 and 33.0% in 2013) and was associated with the highest mortality (30.5% in 2012 and 28.9% in 2013).

The set of laboratory test results available from PHC4 along with their plausible ranges and final categorizations are shown in Additional file 1: Table S2 and S3. Based on BIC criteria, 19 of these laboratory test results were included in the final risk-adjustment model. These tests along with the proportion of results that were normal, abnormal, or missing, are shown in Table 2. For individual laboratory values, the percent of patients with a reported value ranged from 4.4 to 72% in 2012 and 5.2 to 74% in 2013. The most frequently reported lab value was serum glucose, and the least frequently reported lab value was serum pro B-type natriuretic peptide.

**Table 2:** Frequency and proportion of normal, abnormal, and missing lab values by year for each lab value included in the model

Laboratory name	2012			2013		
	Normal	Abnormal	Missing	Normal	Abnormal	Missing
Arterial Bicarbonate	16,406 (14%)	4784 (4%)	94,023 (82%)	17,847 (15%)	5024 (4%)	98,070 (81%)
Arterial pO2	16,735 (15%)	5792 (5%)	92,686 (80%)	17,794 (15%)	6160 (5%)	96,987 (80%)
Arterial SaO2	14,468 (13%)	6602 (6%)	94,143 (82%)	15,371 (13%)	7107 (6%)	98,463 (81%)
Albumin	16,926 (15%)	41,627 (36%)	56,660 (49%)	17,986 (15%)	43,728 (36%)	59,227 (49%)
Alkaline phosphatase	34,812 (30%)	24,172 (21%)	56,229 (49%)	37,344 (31%)	25,706 (21%)	57,891 (48%)
Aspartate aminotransferase	44,493 (39%)	13,969 (12%)	56,751 (49%)	47,748 (39%)	14,829 (12%)	58,364 (48%)
Total bilirubin	42,329 (37%)	15,850 (14%)	57,034 (50%)	46,240 (38%)	16,906 (14%)	57,795 (48%)
Brain natriuretic peptide	13,139 (11%)	4818 (4%)	97,256 (84%)	13,673 (11%)	4910 (4%)	102,358 (85%)
Blood urea nitrogen	23,893 (21%)	58,085 (50%)	33,235 (29%)	26,047 (22%)	61,874 (51%)	33,020 (27%)
Calcium	19,600 (17%)	58,582 (51%)	37,031 (32%)	22,125 (18%)	61,446 (51%)	37,370 (31%)
Creatinine	30,257 (26%)	52,239 (45%)	32,717 (28%)	33,170 (27%)	55,014 (45%)	32,757 (27%)
Glucose	23,536 (20%)	59,543 (52%)	32,134 (28%)	25,418 (21%)	63,569 (53%)	31,954 (26%)
Hemoglobin	12,915 (11%)	69,258 (60%)	33,040 (29%)	14,325 (12%)	73,826 (61%)	32,790 (27%)
International Normalized Ratio	21,267 (18%)	35,534 (31%)	58,412 (51%)	22,790 (19%)	36,892 (31%)	61,259 (51%)
Platelet count	54,541 (47%)	26,501 (23%)	34,171 (30%)	59,165 (49%)	27,908 (23%)	33,868 (28%)
Potassium	53,433 (46%)	28,647 (25%)	33,133 (29%)	58,635 (48%)	29,196 (24%)	33,110 (27%)
Pro-brain natriuretic peptide	868 (1%)	4169 (4%)	110,176 (96%)	1102 (1%)	5160 (4%)	114,679 (95%)
Sodium	45,601 (40%)	37,179 (32%)	32,433 (28%)	48,128 (40%)	40,281 (33%)	32,532 (27%)
Troponin	5050 (4%)	45,168 (39%)	64,995 (56%)	5233 (4%)	48,202 (40%)	67,506 (56%)

The final base model results are shown in Additional file 1: Table S4. The factors most strongly associated with mortality included age, selected hierarchical infection categories (e.g., septicemia, heart infection, lung infection, and fungal infection), and selected comorbidities (e.g., metastatic cancer and neurologic decline). Regarding laboratory results, derangements in pro-BNP, albumin, troponin, bilirubin, BUN, and sodium were most strongly associated with mortality. All models showed good discrimination and calibration. The C-statistics were 0.776 for the 2012 administrative model, 0.772 for the 2013 administrative model, and 0.796 for the 2012 clinical model. The slope and intercept of the calibration plots were 0.960 and 0.007 for the 2012 administrative model, 0.960 and 0.007 for the 2013 administrative model, and 0.965 and 0.006 for the 2012 clinical model.

### Risk-adjusted mortality rates

Risk-adjusted mortality rates varied widely in all models, demonstrating their utility in identifying high performing and low performing hospitals. The range of hospital-specific risk-standardized mortality rates was 12.2 to 24.5% with a mean of 18.4% in the 2012 administrative model; 12.7 to 23.7% with a mean of 18.1% in the 2013 administrative model; and 12.9 to 23.9% with a mean of 18.4% in the 2012 clinical model that included both administrative variables and laboratory results.

**Fig. 3:**
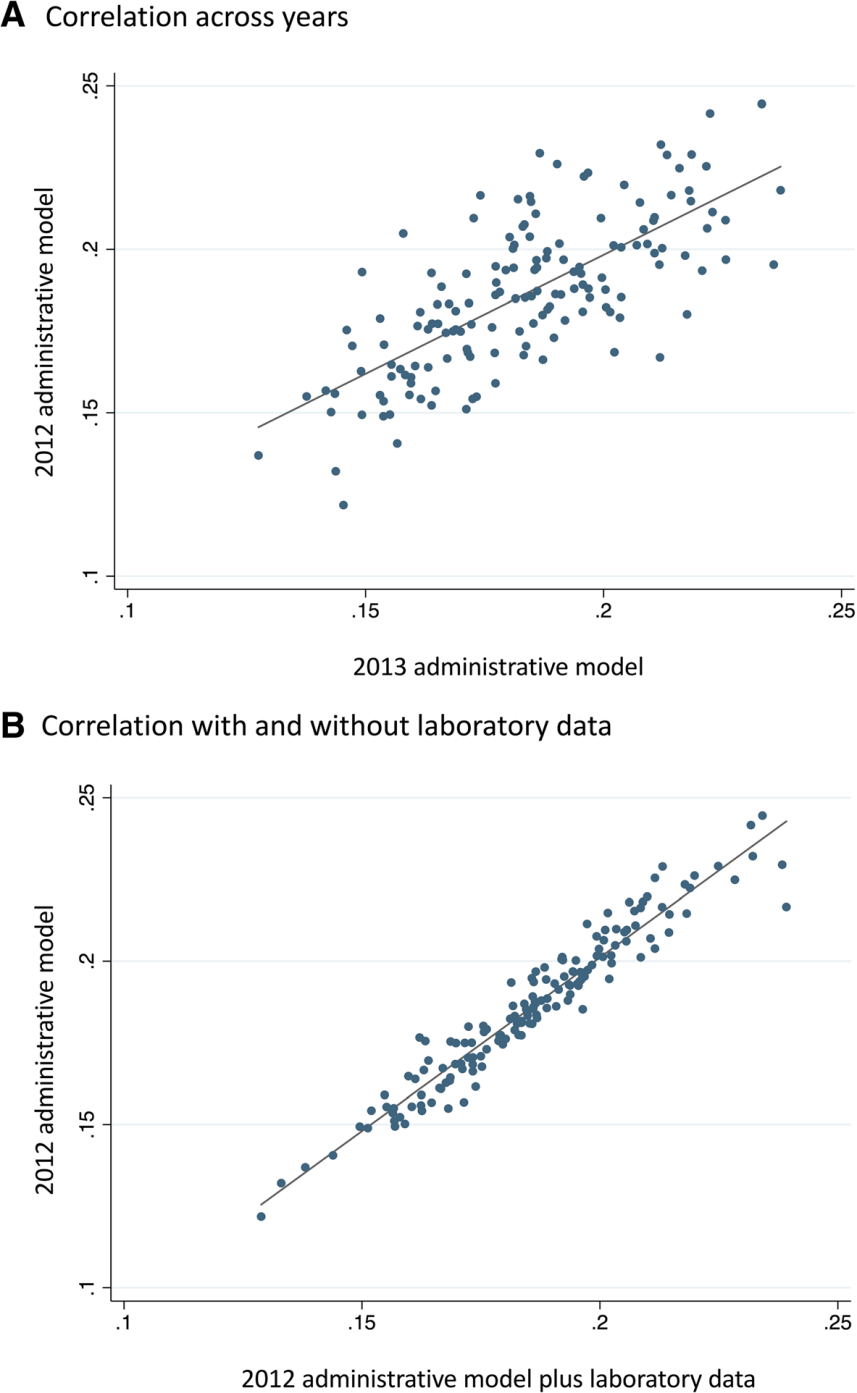
Correlation of risk-adjusted mortality rates between the 2012 and 2013 administrative models (Panel A) and the 2012 administrative and clinical models (Panel B). Y and X axes are the model-derived risk-adjusted mortality rates. Blue dots represent a single hospital. Grey lines represent the linear correlation between the two performance estimates

In the validation steps, the risk-standardized mortality rates for individual hospitals were relatively stable across years (Pearson’s correlation = 0.53; Fig. 3a); and after the addition of laboratory values (Pearson’s correlation = 0.93, Fig. 3b). When stratifying hospitals into quintiles by performance and comparing the 2012 and 2013 administrative models, of the 152 hospitals, 69 (45%) did not change quintile and only 19 (13%) moved by more than one quintile between the 2 years (Table 3**)**. Comparing the 2012 administrative model to the 2012 clinical model, 113 (74%) hospitals stayed in the same quintile, and only 1 hospital (1%) moved by more than one quintile (Table 3).

**Table 3:** Comparison of quintile rankings of individual hospitals by model. Hospitals along the diagonal did not change rankings in the different models, indicating that for these hospitals the performance rankings were stable across time or after the addition of laboratory values

Performance quintile	2012 administrative model					
	1	2	3	4	5	Total
2013 administrative model						
1	18	10	2	1	0	31
2	4	8	14	4	0	30
3	7	10	7	5	1	30
4	2	0	7	14	7	30
5	0	2	1	5	22	30
2012 administrative model + labs						
1	26	5	2	1	0	31
2	5	18	7	0	0	30
3	0	6	22	3	0	31
4	0	1	2	22	5	30
5	0	0	0	5	25	30
Total						
	31	30	31	30	30	152

## Discussion

Using a large, state-wide sample of sepsis admissions to over 150 hospitals, we developed an administrative risk-adjustment model suitable for benchmarking hospitals on their 30-day sepsis mortality. This model showed very good discrimination and calibration. In addition, the model results were reasonably stable, yielding performance assessments that were similar when comparing multiple years and when comparing the administrative model to a model that contained more granular clinical risk adjustment variables.

Our model can be used by health systems and governments to assess hospital performance in the care of patients with sepsis. Sepsis is increasingly recognized as a major public health problem, and there is increasing attention to implementing large-scale sepsis performance improvement initiatives in hospitals [17]. For example, in the United States, the federal government requires all hospitals participating in the Medicare program to report data on adherence to a sepsis care bundle [6]. In addition, several US localities require hospitals to implement protocols for sepsis recognition and treatment [7]. Our model can be used to assess the impact of those initiatives and others like them, providing a valuable tool for sepsis-focused health policy assessment and population-based comparative effectiveness research.

Similarly, our model could allow researchers and policy makers to identify hospitals with outlying performance as candidates for targeted quality improvement efforts. For example, poor performing hospitals could benefit from dedicated resources to improve sepsis outcomes, and high performing hospitals could serve as laboratories to understand how to deliver high-quality sepsis care. This framework, known as “positive-negative deviance” [18], is an increasingly common quality improvement tool and has been useful in other analogous areas such as performance improvement in intensive care unit telemedicine [19].

The current study builds off prior work in this area, including related studies performed in Germany [20], in the United States Medicare population [21], and in patients with septic shock [22]. Our study adds to this literature in that it examined all hospitalized sepsis patients in a large US state and included patients with all insurance types instead of just Medicare, thus filling an important niche. Our study also extends related work which developed an administrative model for sepsis mortality but for which the time horizon was limited to the hospital [23] (i.e. patients were not followed for their outcome after discharge). In-hospital mortality as an outcome measure is known to be biased by discharge practices [24]. Benchmarking hospitals using in-hospital mortality might incentivize them to discharge patients more quickly to post-acute care hospitals, biasing the performance assessments [25, 26]. This problem is overcome when using 30-day mortality as an outcome measure, as we do here, making our results particularly useful.

Our study has several limitations. First, by using administrative data, we cannot rule out that we insufficiently accounted for variation in case-mix across hospitals. Although our comparison to a model that included lab values provides important construct validity, we did not have access to other key variables like vital signs or patients’ preferences for limitations of life-sustaining treatment [27]. Including these values might demonstrate that a more accurate model would perform differently than our administrative model and result in more significant changes in hospital performance rankings. Second, in addition to administrative risk adjustment we used an administrative case-ascertainment strategy, which is only modestly accurate and may lead to different performance rankings than a different administrative strategy or a clinical strategy [28]. Third, we used data from only one US state, however it is a large state with both urban and rural areas, supporting the generalizability of our results. Finally, we examined 30-day mortality but not other important outcome measures like sepsis readmission rates or long-term outcomes. Future work should be directed at understanding hospital-level variation in these outcome measures.

## Conclusions

In conclusion, we developed a robust risk-adjustment model that may be implemented on existing data collection structures and can be used to benchmark hospitals on sepsis outcomes. Future work should be directed at using this model to develop and test large scale sepsis performance improvement initiatives.

## Additional file

Additional file 1: Supplementary Data. Supplementary data referenced in primary manuscript (DOCX 49 kb)

## Abbreviations

BIC: Bayesian information criterion
ICD-9-CM: International Classification of Diseases, Version 9.0—Clinical Modification
PHC4: Pennsylvania Health Care Cost Containment Council
