# Supplementary material for: An administrative model for benchmarking hospitals on their 30-day sepsis mortality
Source: BMC Health Serv Res. 2019 Apr 11;19:221. doi: 10.1186/s12913-019-4037-x (PMC6458755; doi:10.1186/s12913-019-4037-x)
Supplement: Supplementary file 1 — Supplementary Data. Supplementary data referenced in primary manuscript (DOCX 49 kb) [file 12913_2019_4037_MOESM1_ESM.docx]

**SUPPLEMENTARY DATA**

**An administrative model for benchmarking hospitals on their 30-day sepsis mortality**

Darby JL, Davis BS, Barbash IJ, Kahn JM

**Table S1.** Hierarchical infection category groupings

| **Category** | **ICD-9-CM codes** |
| --- | --- |
| Septicemia | 038, 78552, 99592 |
| Heart infection | 420, 421 |
| Peritoneal infection | 00845, 567, 56983 |
| Lung infection | 010, 011, 012, 0310, 481, 482, 485, 486 |
| Fungal infection | 1120, 1124, 1125, 114, 115, 116, 117, 118 |
| Bacteremia | 018, 0312, 7907 |
| Central nervous system infection | 013, 036, 094, 320, 322, 325, 3240, 3241, 3249 |
| Other infection | 001, 002, 0045, 005, 008 (except 00845), 009, 020, 021, 022, 023, 024, 025, 026, 027, 0318, 0319, 032, 033, 034, 037, 039, 040, 041, 090, 091, 092, 093, 095, 096, 097, 100, 101, 102, 103, 104, 49121, 494, 510, 513, 730, 9966, 9985, 9993 |
| Genitourinary infection | 016, 098, 590, 597, 5990, 601, 614, 615, 616 |
| Soft tissue infection | 015, 017, 0311, 035, 451, 110, 111, 1123, 681, 682, 683, 686, 7110, 730 |
| Gastrointestinal infection | 003, 014, 540, 541, 542, 56201, 56203, 56211, 56213, 566, 5695, 5720, 5721, 5750 |
| Upper respiratory infection | 461, 462, 463, 464, 465 |

**Table S2.** Laboratory tests reported to the Pennsylvania Health Care Cost Containment Council. Values are for 2012 only.

| **Test Name** | **Unit** | **Non-missing (%)** | **Mean** | **Plausible low value** | **Plausible high value** |
| --- | --- | --- | --- | --- | --- |
| White blood cell count | 10^9/L | 71% | 12.5 | 0 | 243 |
| Hemoglobin | g/dL | 71% | 11.5 | 2 | 24.6 |
| Platelets | 10^9/L | 70% | 211.2 | 0 | 1734 |
| Neutrophils Band | % | 21% | 12.0 | 0 | 94.4 |
| Prothrombin time | Sec | 46% | 17.5 | 5 | 75 |
| International Normalized Ratio | Ratio | 49% | 1.7 | 0 | 19.7 |
| Partial thromboplastin time | Sec | 41% | 37.2 | 15 | 200 |
| Sodium | mEq/L | 72% | 137.5 | 81 | 188 |
| Potassium | mEq/L | 71% | 4.3 | 0.6 | 10 |
| Glucose | mg/dL | 72% | 151.6 | 20 | 1392 |
| Blood Urea Nitrogen | mg/dL | 71% | 36.6 | 1 | 276 |
| Creatinine | mg/dL | 71% | 1.9 | 0 | 20 |
| Albumin | g/dL | 51% | 3.1 | 0 | 11.3 |
| Calcium | mg/dL | 68% | 8.7 | 5 | 19.6 |
| Total Bilirubin | mg/dL | 50% | 1.2 | 0 | 49.1 |
| Aspartate Aminotransferase | U/L | 51% | 83.2 | 1 | 13789 |
| Alkaline Phosphatase | U/L | 51% | 115.9 | 10.9 | 4308 |
| Creatine Kinase | U/L | 30% | 463.6 | 10 | 49810 |
| Creatine Kinase MB | ng/mL | 22% | 12.2 | 0 | 5512 |
| Troponin I | ng/mL | 36% | 1.0 | 0 | 100 |
| Troponin T | μg/L | 8% | 0.3 | 0 | 56.0 |
| Brain Natriuretic Peptide | pg/mL | 16% | 690.6 | 0 | 9889 |
| pro-BNP | pg/mL | 4% | 7604 | 0 | 100000 |
| Arterial Base Units | mEq/L | 18% | -1.0 | -33.6 | 25.0 |
| Arterial Bicarbonate | mEq/L | 18% | 23.8 | 0 | 100 |
| Arterial SaO2 | % | 18% | 91.6 | 20 | 100 |
| Arterial pH | (none) | 20% | 7.34 | 6 | 8 |
| Arterial pCO2 | mmHg | 20% | 45.7 | 5 | 189 |
| Arterial pO2 | mmHg | 19% | 105.7 | 0 | 639 |

**Table S3.** Cut-points used to categorize the laboratory values. Cut-points were determined from previously published reports and visual inspection of locally weighted scatterplot smoothing plots for 30-day mortality.

| **Test Name** | **Very low** | **Low** | **Normal** | **High** | **Very high** |
| --- | --- | --- | --- | --- | --- |
| White blood cell count |  | ≤4 | >4 ≤10 | >10 ≤20 | >20 |
| Hemoglobin | ≤10 | >10 ≤13.5 | >13.5 |  |  |
| Platelets | ≤100 | >100 ≤150 | >150 ≤450 | >450 |  |
| Neutrophils Band |  |  | ≤5 | >5 <45 | >45 |
| Prothrombin time |  |  | ≤15 | >15 ≤30 | >30 |
| INR |  |  | ≤1.1 | >1.1 ≤3 | >3 |
| PTT |  | ≤25 | >25 ≤35 | >35 ≤100 | >100 |
| Sodium | ≤120 | >120 ≤135 | >135 ≤145 | >145 ≤150 | >150 |
| Potassium |  | ≤2 | >2 ≤4.5 | >4.5 ≤5.5 | >5.5 |
| Glucose |  | ≤70 | >70 ≤120 | >120 ≤400 | >400 |
| Blood Urea Nitrogen |  |  | ≤20 | >20 ≤60 | >60 |
| Creatinine |  | ≤.6 | >.6 ≤1.3 | >1.3 ≤6 | >6 |
| Albumin | ≤2 | >2 ≤3.5 | >3.5 |  |  |
| Calcium | ≤7 | >7 ≤9 | >9 |  |  |
| Total Bilirubin |  |  | ≤1 | >1 ≤4 | >4 |
| Aspartate Aminotransferase |  |  | ≤50 | >50 ≤400 | >400 |
| Alkaline Phosphatase |  |  | ≤100 | >100 ≤250 | >250 |
| Creatine Kinase |  | ≤50 | >50 ≤400 | >400 ≤2.5K | >2.5K |
| Creatine Kinase MB |  |  | ≤5 | >5 ≤150 | >150 |
| Troponin I |  |  | 0 | >0 ≤5 | >5 |
| Troponin T |  |  | ≤.01 | >.01 ≤1 | >1 |
| Brain Natriuretic Peptide |  | ≤100 | >100 ≤2.5K | >2.5K |  |
| pro-BNP |  |  | ≤500 | >500 ≤30K | >30K |
| Arterial Base Units | ≤-20 | >-20 ≤-10 | >-10 ≤10 | >10 |  |
| Arterial Bicarbonate |  | ≤15 | >15 ≤35 | >35 |  |
| Arterial SaO2 | ≤80 | >80 ≤92 | >92 |  |  |
| Arterial pH |  | ≤7.25 | >7.25 |  |  |
| Arterial pCO2 |  | ≤30 | >30 ≤50 | >50 |  |
| Arterial pO2 |  | ≤50 | >50 ≤150 | >150 |  |

**Table S4.**  Full model coefficients for the three final models, with 95% confidence intervals. Coefficients are interpreted as the log-odds of 30-day mortality for each category.

|  | **2012 Model** | **2013 Model** | **2012 Model + lab values** |
| --- | --- | --- | --- |
| **Demographics** |  |  |  |
| Age ≤57 | 0.03 | 0.03 | 0.03 |
|  | (0.03 - 0.03) | (0.03 - 0.04) | (0.02 - 0.03) |
| Age >57 and ≤ 68 | 0.03 | 0.03 | 0.04 |
|  | (0.03 - 0.04) | (0.02 - 0.04) | (0.03 - 0.04) |
| Age >68 and ≤ 77 | 0.04 | 0.04 | 0.03 |
|  | (0.03 - 0.04) | (0.03 - 0.05) | (0.03 - 0.04) |
| Age > 77 and ≤ 85 | 0.04 | 0.04 | 0.05 |
|  | (0.04 - 0.05) | (0.04 - 0.05) | (0.04 - 0.05) |
| Age > 85 | 0.07 | 0.06 | 0.07 |
|  | (0.06 - 0.08) | (0.05 - 0.07) | (0.06 - 0.09) |
| Female | -0.06 | -0.06 | -0.07 |
|  | (-0.09 - -0.02) | (-0.09 - -0.03) | (-0.11 - -0.04) |
| **Admission through ED** | 0.01 | 0.03 | 0.05 |
|  | (-0.04 - 0.05) | (-0.01 - 0.08) | (0.01 - 0.10) |
| **Comorbidities** |  |  |  |
| Congestive heart failure | 0.27 | 0.25 | 0.24 |
|  | (0.23 - 0.31) | (0.21 - 0.29) | (0.20 - 0.28) |
| Valvular disease | -0.05 | -0.1 | -0.05 |
|  | (-0.12 - 0.01) | (-0.16 - -0.04) | (-0.11 - 0.02) |
| Pulmonary circulation disease | 0.25 | 0.27 | 0.23 |
|  | (0.18 - 0.32) | (0.21 - 0.34) | (0.16 - 0.30) |
| Peripheral vascular disease | 0.19 | 0.23 | 0.17 |
|  | (0.13 - 0.24) | (0.17 - 0.28) | (0.12 - 0.23) |
| Paralysis | 0.09 | 0.1 | 0.06 |
|  | (0.01 - 0.16) | (0.02 - 0.17) | (-0.02 - 0.14) |
| Neurological disorder | 0.11 | 0.02 | 0.11 |
|  | (0.06 - 0.16) | (-0.03 - 0.06) | (0.06 - 0.16) |
| Chronic pulmonary disease | 0.11 | 0.1 | 0.14 |
|  | (0.07 - 0.15) | (0.06 - 0.13) | (0.10 - 0.18) |
| Diabetes w/o chronic complications | -0.07 | -0.05 | -0.06 |
|  | (-0.11 - -0.03) | (-0.09 - -0.01) | (-0.10 - -0.02) |
| Diabetes w/ chronic complications | -0.14 | -0.19 | -0.14 |
|  | (-0.20 - -0.07) | (-0.26 - -0.13) | (-0.22 - -0.07) |
| Hypothyroidism | -0.1 | -0.05 | -0.09 |
|  | (-0.15 - -0.06) | (-0.10 - -0.01) | (-0.14 - -0.04) |
| Renal failure | 0.15 | 0.14 | 0.14 |
|  | (0.12 - 0.19) | (0.11 - 0.18) | (0.10 - 0.18) |
| Liver disease | 0.5 | 0.5 | 0.38 |
|  | (0.43 - 0.58) | (0.43 - 0.58) | (0.30 - 0.46) |
| Lymphoma | 0.43 | 0.41 | 0.44 |
|  | (0.32 - 0.55) | (0.30 - 0.52) | (0.32 - 0.56) |
| Metastatic cancer | 1.42 | 1.41 | 1.38 |
|  | (1.36 - 1.49) | (1.35 - 1.48) | (1.31 - 1.45) |
| Solid tumor w/o metastasis | 0.52 | 0.52 | 0.49 |
|  | (0.44 - 0.60) | (0.44 - 0.59) | (0.41 - 0.57) |
| Rheumatoid arthritis | -0.02 | -0.04 | 0.02 |
|  | (-0.12 - 0.07) | (-0.13 - 0.05) | (-0.08 - 0.12) |
| Coagulopathy | 0.24 | 0.16 | 0.18 |
|  | (0.17 - 0.30) | (0.09 - 0.23) | (0.11 - 0.25) |
| Obesity | -0.23 | -0.22 | -0.21 |
|  | (-0.29 - -0.17) | (-0.28 - -0.16) | (-0.27 - -0.15) |
| Weight Loss | 0.38 | 0.34 | 0.3 |
|  | (0.34 - 0.43) | (0.29 - 0.38) | (0.26 - 0.35) |
| Fluid and electrolyte disorder | 0.22 | 0.25 | 0.18 |
|  | (0.18 - 0.25) | (0.21 - 0.29) | (0.14 - 0.22) |
| Chronic blood loss anemia | -0.21 | -0.1 | -0.28 |
|  | (-0.36 - -0.05) | (-0.25 - 0.05) | (-0.44 - -0.12) |
| Deficiency anemia | -0.17 | -0.19 | -0.17 |
|  | (-0.20 - -0.13) | (-0.23 - -0.15) | (-0.21 - -0.13) |
| Alcohol abuse | 0.02 | -0.06 | -0.05 |
|  | (-0.07 - 0.12) | (-0.15 - 0.03) | (-0.14 - 0.05) |
| Drug abuse | -0.43 | -0.4 | -0.39 |
|  | (-0.58 - -0.28) | (-0.54 - -0.26) | (-0.55 - -0.23) |
| Psychotic disorder | -0.37 | -0.32 | -0.35 |
|  | (-0.45 - -0.29) | (-0.40 - -0.24) | (-0.44 - -0.27) |
| Depression | -0.12 | -0.12 | -0.09 |
|  | (-0.17 - -0.06) | (-0.18 - -0.07) | (-0.14 - -0.03) |
| Hypertension | -0.24 | -0.29 | -0.2 |
|  | (-0.28 - -0.21) | (-0.32 - -0.25) | (-0.24 - -0.17) |
| **Organ failures present-on-admission** |  |  |  |
| Septic shock | 0.43 | 0.56 | 0.31 |
|  | (0.36 - 0.51) | (0.49 - 0.63) | (0.23 - 0.38) |
| Respiratory failure | 0.78 | 0.76 | 0.67 |
|  | (0.74 - 0.82) | (0.71 - 0.80) | (0.62 - 0.72) |
| Cardiovascular failure | -0.06 | -0.1 | -0.1 |
|  | (-0.11 - -0.00) | (-0.16 - -0.05) | (-0.15 - -0.04) |
| Renal failure | -0.1 | -0.17 | -0.19 |
|  | (-0.13 - -0.06) | (-0.21 - -0.14) | (-0.23 - -0.15) |
| Hepatic failure | 0.96 | 0.97 | 0.66 |
|  | (0.87 - 1.05) | (0.88 - 1.06) | (0.56 - 0.76) |
| Hematologic failure | -0.15 | -0.14 | -0.17 |
|  | (-0.23 - -0.07) | (-0.22 - -0.06) | (-0.25 - -0.09) |
| Metabolic failure | 0.37 | 0.35 | 0.25 |
|  | (0.32 - 0.42) | (0.30 - 0.40) | (0.20 - 0.31) |
| Neurologic failure | 1.03 | 0.97 | 0.99 |
|  | (0.93 - 1.13) | (0.87 - 1.07) | (0.89 - 1.10) |
| **Hierarchical infection category** |  |  |  |
| Septicemia | 1.94 | 1.8 | 1.89 |
|  | (1.58 - 2.30) | (1.46 - 2.14) | (1.52 - 2.25) |
| Heart infection | 1.47 | 1.58 | 1.42 |
|  | (1.04 - 1.90) | (1.17 - 1.99) | (0.99 - 1.85) |
| Peritoneal infection | 1.28 | 1.21 | 1.25 |
|  | (0.90 - 1.65) | (0.86 - 1.56) | (0.88 - 1.62) |
| Lung infection | 1.42 | 1.34 | 1.42 |
|  | (1.06 - 1.79) | (1.00 - 1.69) | (1.06 - 1.79) |
| Fungal infection | 1.47 | 1.36 | 1.45 |
|  | (1.09 - 1.86) | (1.00 - 1.72) | (1.07 - 1.83) |
| Bacteremia | 0.94 | 0.92 | 0.93 |
|  | (0.56 - 1.33) | (0.55 - 1.29) | (0.54 - 1.32) |
| Central nervous system infection | 1.36 | 1.19 | 1.38 |
|  | (0.85 - 1.86) | (0.71 - 1.68) | (0.88 - 1.89) |
| Other infection | 0.8 | 0.71 | 0.79 |
|  | (0.43 - 1.16) | (0.36 - 1.05) | (0.43 - 1.16) |
| Genitourinary infection | 0.88 | 0.91 | 0.85 |
|  | (0.51 - 1.25) | (0.57 - 1.26) | (0.49 - 1.22) |
| Soft tissue infection | 0.64 | 0.73 | 0.6 |
|  | (0.27 - 1.02) | (0.37 - 1.08) | (0.22 - 0.97) |
| Gastrointestinal infection | 0.42 | 0.33 | 0.46 |
|  | (-0.01 - 0.84) | (-0.07 - 0.73) | (0.04 - 0.89) |
| **Laboratory Values** |  |  |  |
| Arterial Blood Gas – pO2 |  |  |  |
| High |  |  | 0.29 |
|  |  |  | (0.20 - 0.38) |
| Low |  |  | 0.08 |
|  |  |  | (-0.08 - 0.23) |
| Arterial Blood Gas - Bicarbonate |  |  |  |
| High |  |  | 0.48 |
|  |  |  | (0.36 - 0.60) |
| Low |  |  | 0.34 |
|  |  |  | (0.24 - 0.43) |
| B-type Natriuretic Peptide |  |  |  |
| High |  |  | 0.6 |
|  |  |  | (0.45 - 0.74) |
| Low |  |  | -0.24 |
|  |  |  | (-0.34 - -0.13) |
| Glucose |  |  |  |
| Very high |  |  | -0.21 |
|  |  |  | (-0.38 - -0.04) |
| High |  |  | -0.15 |
|  |  |  | (-0.20 - -0.11) |
| Low |  |  | 0.17 |
|  |  |  | (0.09 - 0.26) |
| Potassium |  |  |  |
| Very high |  |  | 0.34 |
|  |  |  | (0.27 - 0.42) |
| High |  |  | 0.19 |
|  |  |  | (0.14 - 0.23) |
| Low |  |  | 0.18 |
|  |  |  | (-0.26 - 0.61) |
| Creatinine |  |  |  |
| Very high |  |  | -0.43 |
|  |  |  | (-0.56 - -0.30) |
| High |  |  | -0.16 |
|  |  |  | (-0.21 - -0.11) |
| Low |  |  | 0.15 |
|  |  |  | (0.05 - 0.25) |
| Blood Urea Nitrogen |  |  |  |
| Very high |  |  | 0.52 |
|  |  |  | (0.45 - 0.60) |
| High |  |  | 0.11 |
|  |  |  | (0.05 - 0.16) |
| Alkaline Phosphatase |  |  |  |
| Very high |  |  | 0.34 |
|  |  |  | (0.24 - 0.44) |
| High |  |  | 0.07 |
|  |  |  | (0.02 - 0.12) |
| Troponin |  |  |  |
| Very high |  |  | 0.53 |
|  |  |  | (0.42 - 0.65) |
| High |  |  | 0.03 |
|  |  |  | (-0.01 - 0.07) |
| Bilirubin |  |  |  |
| Very high |  |  | 0.54 |
|  |  |  | (0.42 - 0.66) |
| High |  |  | -0.03 |
|  |  |  | (-0.09 - 0.02) |
| Pro B-type Natriuretic Peptide |  |  |  |
| Very high |  |  | 0.82 |
|  |  |  | (0.57 - 1.08) |
| High |  |  | 0.05 |
|  |  |  | (-0.04 - 0.14) |
| Aspartate Aminotransferase |  |  |  |
| Very high |  |  | 0.14 |
|  |  |  | (0.01 - 0.28) |
| High |  |  | 0.15 |
|  |  |  | (0.09 - 0.20) |
| Platelets |  |  |  |
| Very low |  |  | 0.17 |
|  |  |  | (0.10 - 0.23) |
| High |  |  | 0.07 |
|  |  |  | (-0.04 - 0.18) |
| Low |  |  | -0.16 |
|  |  |  | (-0.22 - -0.11) |
| Hemoglobin |  |  |  |
| Very low |  |  | -0.04 |
|  |  |  | (-0.10 - 0.01) |
| Low |  |  | -0.25 |
|  |  |  | (-0.30 - -0.20) |
| Albumin |  |  |  |
| Very low |  |  | 0.79 |
|  |  |  | (0.71 - 0.88) |
| Low |  |  | 0.12 |
|  |  |  | (0.08 - 0.17) |
| Calcium |  |  |  |
| Very low |  |  | -0.33 |
|  |  |  | (-0.42 - -0.23) |
| Low |  |  | -0.29 |
|  |  |  | (-0.34 - -0.25) |
| Arterial Blood Gas – O2 Saturation |  |  |  |
| extra low |  |  | 0.2 |
|  |  |  | (0.04 - 0.37) |
| Low |  |  | 0.28 |
|  |  |  | (0.20 - 0.36) |
| Sodium |  |  |  |
| Very high |  |  | 0.56 |
|  |  |  | (0.45 - 0.66) |
| Very low |  |  | 0.18 |
|  |  |  | (-0.02 - 0.38) |
| High |  |  | 0.28 |
|  |  |  | (0.19 - 0.37) |
| Low |  |  | -0.13 |
|  |  |  | (-0.17 - -0.08) |
| International Normalized Ratio |  |  |  |
| Very high |  |  | 0.34 |
|  |  |  | (0.26 - 0.42) |
| High |  |  | 0.18 |
|  |  |  | (0.14 - 0.22) |
|  |  |  |  |
| **Regression constant** | -5.64 | -5.66 | -5.48 |
|  | -6.08 - -5.20 | -6.08 - -5.24 | -5.93 - -5.04 |
